# Human-wildlife interaction: past, present, and future

**DOI:** 10.1186/s40850-023-00168-7

**Published:** 2023-05-11

**Authors:** Edward Narayan, Naureen Rana

**Affiliations:** 1 School of Agriculture and Food Sciences, The University of Queensland, Gatton, QLD 4343 Australia; 2 Departmet of Zoology, Wildlife and Fisheries, University of Agriculture Faisalabad, Faisalabad, Pakistan

## Abstract

Human-wildlife interaction is a broad and complex topic. Due to rapid world population growth, there have been greater human impacts on wildlife through agriculture and land fragmentation. In many countries, significant challenges exist with managing wildlife and its negative impacts on humans and wildlife. This special issue discusses human-wildlife co-existence.

Humans have lived alongside animals, especially wildlife since humanity’s humble beginnings as depicted in the historical scripts of many ancient cultures around the world. For example, Aboriginal Australians have coexisted with megafauna for at least 17,000 years. Wildlife such as Dingos were tamed by Aboriginal Australians and used as companion animals [1]. This strong connection between indigenous communities, the land and its wildlife has been acknowledged by the scientific community and strategies of conservation organisations are increasingly applying indigenous knowledge to wildlife conservation approaches globally. The World Wildlife Fund (WWF) defines human-wildlife interaction as a neutral term referring to any encounter between people and wildlife [2]. Human-wildlife conflict (HWC) refers to struggles that arise when the presence or behaviour of wildlife poses actual or perceived direct, recurring threats to human interests or needs, often leading to disagreements between groups of people and negative impacts on people and/or wildlife [2].

Wildlife species are facing ever-increasing pressures to co-exist with humans today. The human population has reached eight billion [3] with increased colonisation of humans in the past century supported with increased advancements in human medicine and landscape expansion for creating space for human dwellings. As human populations continue to grow and expand into the natural habitats of wild animals, the frequency of interactions between humans and animals increases. This human impact on natural ecosystems has resulted in habitat loss and a decline in biodiversity.

Additionally, wildlife can cause damage to livestock and crops, leading to loss of property and livelihood. As a result, measures have been put in place to manage and conserve wildlife to reduce these conflicts. The major human activities that lead to wildlife mortality include hunting, agriculture, pollution, the prevalence of livestock husbandry or shepherding, road traffic, institutional factors such as legal frameworks (e.g., wildlife hunting bans which tends to increase population of wildlife within limited habitat space) and the attitude and tolerance of people living in the area.

There are many examples of conflicts between humans and wild animals, however, the most significant ones are those where both animals and humans become harmful to each other’s existence.

For example, snow leopards in Asia have faced significant impacts by livestock farming, leading to the loss of wildlife habitat. Since snow leopards prey on livestock, farmers often kill the leopards to protect their property. Research conducted in China, India, Nepal and Pakistan has shown that compensation programs and livestock management strategies are highly successful and are recommended to mitigate human-snow leopard conflict. Researchers have also recommended the re-consideration of rangeland management and a better evaluation of the efficacy of existing measures to mitigate human snow-leopard conflict, taking into account the ecological and socio-cultural context of such conflicts [4]. In addition, wildlife species that have become feral animals, such as wild pigs, are also major contributors to human-wildlife conflict in Asian countries [5]. The bird community in South Asian regions, particularly in Pakistan, India, Nepal, Bangladesh and others, have been facing numerous challenges. These challenges have contributed to a decline in their population, which could have impact on ecological services [6, 7].

Keystone and apex predatory wildlife species in Europe are in decline. Interactions between humans with large carnivores such as the brown bear and Iberian lynx are increasing as wildlife species attempt to recolonise their decimated landscapes. However, human pressure on habitat availabilitycontinues to impact the recolonisation of these animals in their natural environment. Humans have also created a social-ecological problem by installing barriers such as railroads and highways, which further hinder the movement of wildlife [8]. In Australia, there is a major ecological disaster in progress due to conflicts between agriculture and mining industries and wildlife conservation. The ever-increasing need for fertile land and water resourceshas resulted in many pristine lands and rivers becoming exposed to developments such as irrigation and livestock grazing, thereby threatening the survival of wildlife [9].

In this special issue, we will explore the important topic of human-animal interactions by inviting papers from researchers worldwide. This will provide a comprehensive and balanced research perspective and enable potential solutions for co-existence of humans and wildlife.

## Data Availability

NA.

## Abbreviations

WWF: World Wildlife Find
HWC: Human-wildlife conflict
